# Histological subtype of gastric adenocarcinoma: two cases of mixed fundic and pyloric mucosa-type adenocarcinoma

**DOI:** 10.3332/ecancer.2020.1143

**Published:** 2020-11-13

**Authors:** Haruhiko Takahashi, Kenshi Yao, Tetsuya Ueo, Takashi Nagahama, Kentaro Imamura, Kenta Chuman, Hiroshi Tanabe, Akinori Iwashita, Toshiharu Ueki

**Affiliations:** 1 Department of Gastroenterology, Fukuoka University Chikushi Hospital, 1-1-1 Zokumyoin Chikushino, Fukuoka 818-8052, Japan; 2 Department of Endoscopy, Fukuoka University Chikushi Hospital, Chikushino, 818-8052, Japan; 3 Department of Gastroenterology, Oita Red Cross Hospital, Oita, 870-0033, Japan; 4 Department of Pathology, Fukuoka University Chikushi Hospital, Chikushino, 818-8052, Japan

**Keywords:** early gastric cancer, gastric phenotype, magnifying narrow-band imaging

## Abstract

Adenocarcinomas with differentiation towards fundic or pyloric glands are rare histological subtypes. We herein describe two cases of new histological subtypes: mixed fundic and pyloric mucosa-type adenocarcinoma detected in *Helicobacter pylori* uninfected patients. The first patient was a woman in her 40s. A glossy, reddish, nodular lesion with a flat elevated whitish area was detected at the gastric fundus. When the nodular lesion was visualised with magnifying narrow-band imaging (M-NBI), an absent microvascular pattern plus an irregular microsurface pattern with a demarcation line was observed. The second patient was a woman in her 60s. A glossy, reddish, elevated lesion was detected at the gastric body. M-NBI finding was a regular microvascular pattern plus a regular microsurface pattern with a demarcation line. Histological examination of the resected specimens from both cases showed a very well- to well-differentiated adenocarcinoma which has differentiation towards the mixed fundic and pyloric mucosa. The histological and serological findings of both cases indicated the absence of *H. pylori* infection. The present two cases demonstrate further evidence of a new histological subtype of gastric adenocarcinoma: mixed fundic and pyloric mucosa-type adenocarcinoma, which has distinct characteristic endoscopic findings.

## Introduction

Gastric cancer usually develops in background mucosa with chronic gastritis associated with *Helicobacter pylori* (*H. pylori*) infection. It is very rare for gastric cancer to develop in the stomach with no evidence of *H. pylori* infection [1]. In recent years, well-differentiated adenocarcinoma with gastric phenotype, such as gastric adenocarcinoma of fundic gland type, has been reported as a new disease entity in the *H. pylori*-negative patient [2]. We herein report the characteristics of endoscopic and histological findings in two *H. pylori*-negative patients as a new histological subtype: mixed fundic and pyloric mucosa-type gastric adenocarcinoma, which has differentiation into pyloric gland-like cells and fundic gland-like cells, respectively.

## Case report

### Case 1

**Figure 1.**
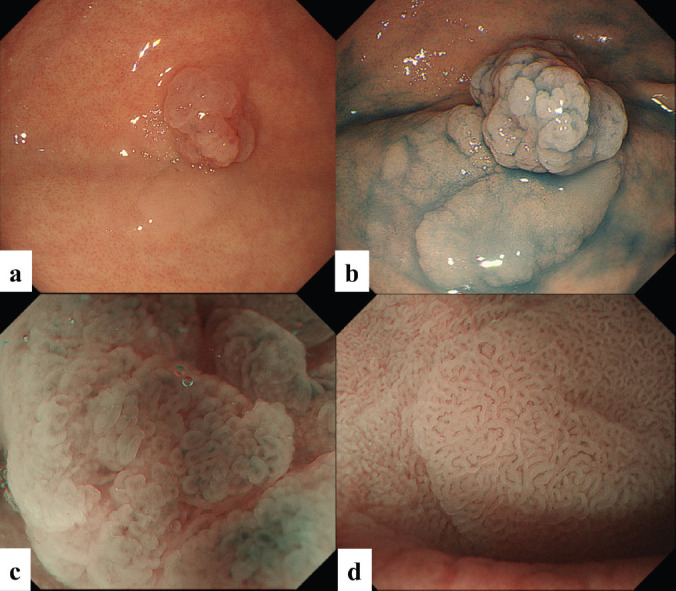
Case 1. Endoscopic findings. (a, b): Endoscopic images by conventional white-light endoscopy. (a): A distinct, glossy, reddish, nodular lesion is present in the greater curvature of the gastric fundus with an accompanying whitish flat elevated area at the base. The margin becomes clearer with indigo carmine dye spraying. The size is approximately 25 mm, and the surface of the lesion consists of nodules of varying sizes. (b): The flat elevated area shows a well-organised, fine granular and villous structure on its surface. (c, d): Endoscopic images by magnifying narrow-band imaging (M-NBI). (c): M-NBI findings of the nodular elevated lesion depict the absence of an MV pattern plus an irregular MS pattern with a demarcation line. A VEC pattern can also be detected. (d). M-NBI findings of the flat elevated area demonstrate a regular MV pattern plus a regular MS pattern with a demarcation line.

**Figure 2.**
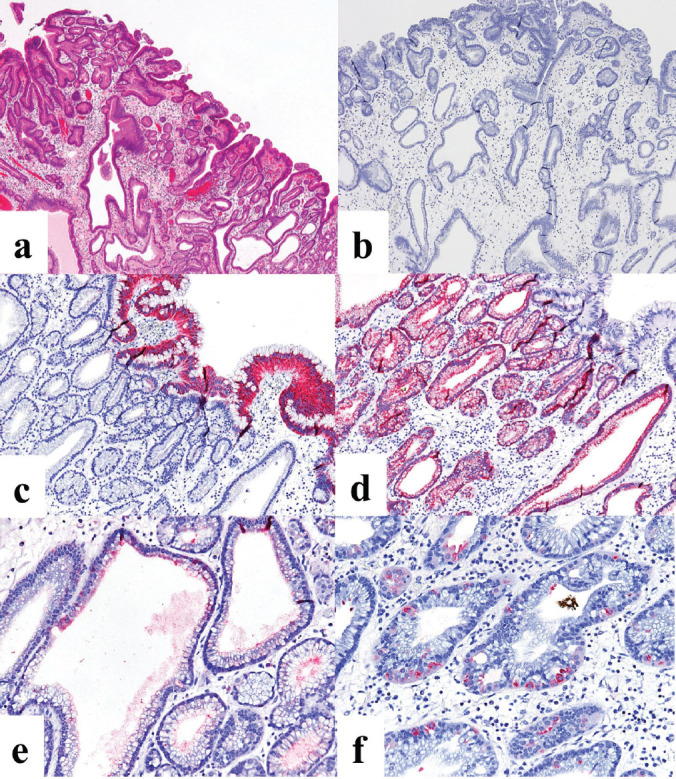
Case 1. Histopathological findings of the resected specimen. (a): Haematoxylin and eosin staining. The nodular part mainly consists of densely proliferating gland ducts of varying sizes and dilated ducts with interstitial oedema. (b-f): Immunohistochemical staining. (b): On immunostaining, the chief cell-like cells are negative for pepsinogen. (c): Surface foveolar epithelium-like cells are positive for MUC5AC. Pyloric gland-like cells are positive for (d): MUC6 and (e): lysozyme. (f): Parietal cell-like cells are positive for proton pumps.

The patient was a woman in her 40s. An elevated lesion had been detected in the greater curvature at the fundus on screening upper gastrointestinal endoscopy. On conventional white-light endoscopy, no atrophic changes were seen in the background mucosa, and there was a regular arrangement of collecting venules (RAC). The serological findings indicated the absence of* H. pylori* infection. A distinct, glossy, reddish, nodular, elevated lesion was detected in the greater curvature of the gastric fundus with a whitish flat elevated area (Figure 1a). The margin of the lesion became clearer after indigo carmine dye spraying, and the lesion was approximately 25 mm in size. (Figure 1b). A clear demarcation line was visualised by magnifying endoscopy with narrow-band imaging (M-NBI). When the nodular elevation was observed using M-NBI according to the VS (vessel plus surface) classification system [3], an absent microvascular pattern plus an irregular microsurface pattern was observed. It was also accompanied by so-called vessels within an epithelial circle (VEC) pattern [4] (Figure 1c). The flat elevated area was classed as a regular microvascular pattern plus a regular microsurface pattern (Figure 1d). Neither light blue crest [5] nor white opaque substance [6], which are the markers for intestinal phenotype, was detected. Well-differentiated adenocarcinoma with gastric phenotype in *H. pylori* negative was suspected. Histopathological examination of the resected specimens revealed that the background mucosa was histologically negative for *H. pylori*. The tumour was diagnosed as very well- to well-differentiated adenocarcinoma localised within the mucosa. The nodular part mainly consists of densely proliferating gland ducts of varying sizes and dilated ducts with interstitial oedema. Papillary structures were also seen at the tumour surface (Figure 2a). The tumour tissue consisted of pyloric gland-like tubular ducts. On immunostaining, the chief cell-like cells are negative for pepsinogen (Figure 2b), surface foveolar epithelium-like cells are positive for MUC5AC (Figure 2c) and pyloric gland-like cells are positive for MUC6 (Figure 2d) and lysozyme (Figure 2e). Parietal cell-like cells are positive for proton pumps (Figure 2f). The final diagnosis was very well- to well-differentiated adenocarcinoma with a gastric phenotype of mixed fundic and pyloric mucosa type, which has differentiation towards pyloric glands and parietal cells, and foveolar epithelium.

### Case 2

**Figure 3.**
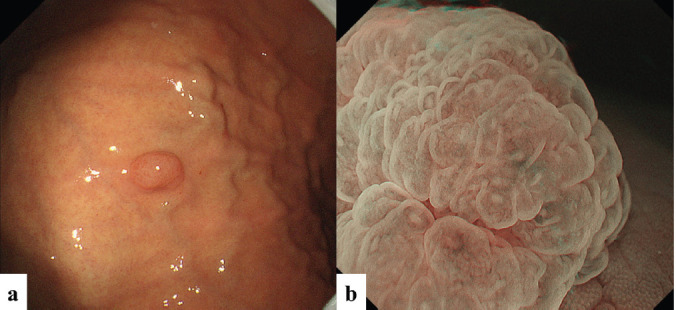
Case 2. Endoscopic findings. (a): Endoscopic images by conventional white-light endoscopy. An elevated lesion, 8 mm in size, can be seen in the gastric body greater curvature. (b): Endoscopic images by M-NBI. M-NBI findings showed a regular MV pattern plus a regular MS pattern with a demarcation line.

**Figure 4.**
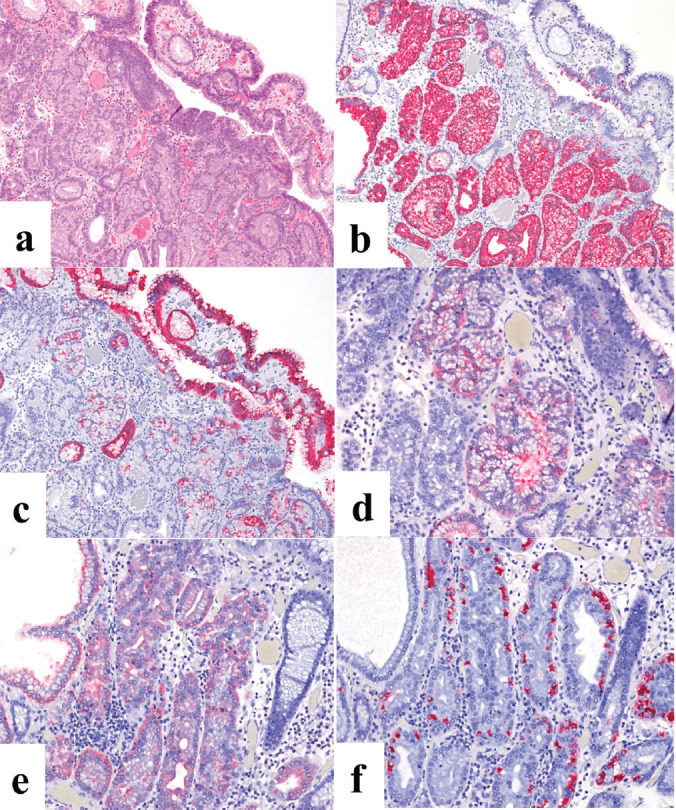
Case 2. Histopathological findings of the resected specimen. (a): Haematoxylin and eosin staining. The tumour consists of densely proliferating tumour ducts with a slightly atypical structure. Dilated tumour ducts are present in some parts. (b-f): Immunohistochemical staining. (b): On immunostaining, the chief cell-like cells are mainly positive for pepsinogen and (c): the foveolar epithelium-like cells are mainly positive for MUC5AC. The mucous neck cell-like cells are mainly positive for MUC6; the pyloric gland-like cells are mainly positive for (d) MUC6 and (e) lysozyme. (f): Parietal cell-like cells are mainly positive for proton pumps.

The second patient was a woman in her 60s. An elevated lesion had been detected on screening endoscopy. On conventional white-light endoscopy, no atrophic changes were seen in the background mucosa, and there was a RAC. The serological findings indicated the absence of* H. pylori* infection. A glossy, slightly reddish nodule measuring 8 mm in diameter was detected in the greater curvature of the gastric body (Figure 3a). M-NBI findings showed a regular MV pattern composed of loop-shaped microvessels and a regular MS pattern with a demarcation line (Figure 3b). Neither light blue crest [5] nor white opaque substance [6], which are the markers for intestinal phenotype, was detected. From the endoscopic findings, it was difficult to determine whether the lesion was gastric cancer or adenoma. Histopathological examination of resected specimens demonstrated a very well- to well-differentiated adenocarcinoma with shallow submucosal invasion (400 μm). Lymphovascular invasion was not detected. The tumour consists of densely proliferating tumour ducts with a slightly atypical structure. Dilated tumour ducts are present in some parts (Figure 4a). On immunostaining, the chief cell-like cells are mainly positive for pepsinogen (Figure 4b), the foveolar epithelium-like cells are mainly positive for MUC5AC (Figure 4c). The mucous neck cell-like cells are mainly positive for MUC6; the pyloric gland-like cells are mainly positive for MUC6 (Figure 4d) and lysozyme (Figure 4e). Parietal cell-like cells are mainly positive for proton pumps (Figure 4f). All tumour cells were negative for MUC2 and CD10. Accordingly, the tumour has a gastric phenotype with differentiation into fundic gland, pyloric gland and foveolar epithelium. The final diagnosis was very well- to well-differentiated adenocarcinoma with a gastric phenotype of mixed fundic and pyloric mucosa type.

## Discussion

Development of gastric cancer in nonatrophic mucosa without *H. pylori* infection is very rare [1]. However, in actual clinical practice, *H. pylori*-negative gastric cancer is occasionally encountered. It has been reported to be about 1% and to comprise a higher rate of undifferentiated gastric cancer (signet-ring cell carcinoma) and a superficial depressed macroscopic appearance [7].

Recently, cases of gastric epithelial neoplasia (adenoma and cancer) with gastric phenotype arising from *H. pylori*-negative mucosa have been reported. Gastric adenocarcinoma of fundic gland type was reported as a new subtype of low-grade adenocarcinoma with gastric phenotype [2, 8]. Furthermore, other variants composed not only of pure gastric adenocarcinoma of fundic gland type but also having a differentiation towards surface foveolar epithelium have been reported. Tanabe et al. reported this new histologic variant as ‘gastric adenocarcinoma fundic mucosa type’ [9]. More recently, Kanesaka et al. reported a case of mixed fundic and pyloric mucosa-type adenocarcinoma which has differentiation towards both pyloric gland-like cells and fundic gland-like cells as one of a new subtype of ‘gastric adenocarcinoma fundic mucosa type’ [10].

The common characteristics of conventional endoscopic findings in the present two cases were that the lesions were glossy, reddish and elevated and that they developed in nonatrophic fundic gland mucosa. However, the endoscopic findings of the lesions clearly differed from those for gastric adenocarcinoma of fundic gland type or gastric adenocarcinoma of fundic mucosa type. Ueyama et al. reported four characteristics for the endoscopic findings in gastric adenocarcinoma of fundic gland type, these being 1) submucosal tumour shape, 2) whitish colour, 3) presence of branched dilated vessels and 4) no atrophic changes in the background surrounding mucosa [8]. In the current two cases, no atrophic changes were present in the background mucosa, but there was faint redness and a steep elevation. These findings clearly differed from the submucosal tumour shape that is characteristic of gastric adenocarcinoma of fundic gland type and gastric adenocarcinoma of fundic mucosa type. On M-NBI, a clear demarcation line with the surrounding mucosa was present. This finding also differed from the M-NBI findings of gastric adenocarcinoma of fundic gland type in that tumour epithelium was not seen on the surface of those lesions. These differences in morphological characteristics suggest that the two cases reported here represent a completely different subtype from that seen in the previously reported cases of gastric adenocarcinoma of fundic gland type or gastric adenocarcinoma of fundic mucosa type. A VEC pattern [4] on the surface of the elevated lesion was also detected in one case by M-NBI, and therefore, adenocarcinoma with a papillary structure was suspected. In fact, a papillary structure was histologically identified in the corresponding specimen. There is only one case report of mixed fundic and pyloric mucosa-type adenocarcinoma reported by Kanesaka *et al* [10]. Compared to this first reported case, the findings of the histology and immunochemistry are almost similar, but the endoscopic images are different from our cases.

In this new histological subtype, tumour cells differentiate into both pyloric and fundic gland-like cells located in the deep tumour area, while tumour cells differentiate into foveolar-like cells located on the surface tumour area. The information obtained by M-NBI is limited in the morphological findings on the surface area composed of foveolar-like tumour cells. This may be the cause of the variability in endoscopic findings of this tumour. However, this explanation is not beyond speculation, and the genetic disorders associated with this new histological subtype are still unknown. Therefore, further studies are needed to clarify these issues.

## Conclusion

The two cases described herein demonstrate further evidence of a new histological subtype of gastric adenocarcinoma. These endoscopic findings may indicate the characteristics of this new subtype.

## Conflicts of interest

The authors declare that they have no conflicts of interest regarding the publication of this paper.

## Funding

The authors did not receive any funding for this work.
